# Face Mask Acceptability for Communal Religious Worship During the COVID-19 Pandemic in the United Kingdom: Results from the CONFESS Study

**DOI:** 10.1007/s10943-022-01641-2

**Published:** 2022-08-24

**Authors:** Kai Man Alexander Ho, Rebecca F. Baggaley, Timothy C. Stone, Áine Hogan, Yusuf Kabir, Christopher Johnson, Robert Merrifield, Laurence B. Lovat

**Affiliations:** 1 Division of Surgery and Interventional Science, University College London, Charles Bell House, 43-45 Foley Street, London, W1W 7TY UK; 2 Wellcome/EPSRC Centre for Interventional and Surgical Sciences (WEISS), University College London, Charles Bell House, 43-45 Foley Street, London, W1W 7TY UK; 3 Department of Respiratory Sciences, University of Leicester, University Road, Leicester, LE1 7RH UK; 4 Dynaimx, 71-75 Shelton Street, London, WC2H 9JQ UK

**Keywords:** COVID-19, Virus transmission, Face masks, Religion, Singing

## Abstract

The COVID-19 pandemic has led to restrictions such as social distancing and mandatory wearing of face masks. Singing and religious gatherings have been linked to infection clusters, and between 2020 and 2021 indoor congregational singing and chanting were prohibited in the United Kingdom. We evaluated attitudes to face mask use and their acceptability as well as changes within places of worship since their reopening in July up to autumn 2020. In this cross-sectional study, participants were recruited using convenience sampling through selective targeting of religious organisations and social media. Participants self-enrolled and completed an online questionnaire, which included open and closed questions. We used multivariable logistic regression to identify factors associated with face mask acceptability. We performed thematic analysis to evaluate responses to open questions. A total of 939 participants were included in the analysis. Median age was 52.7 years and 66.1% were female, while 80.7% identified as Christian. A majority (672/861; 78.0%) of participants would find it acceptable to wear a face mask and reduce their singing or chanting volume if required, even though 428/681 (49.1%) found face masks to be uncomfortable. Multivariable regression found that younger age was associated with a higher acceptability of face masks (adjusted OR (aOR): 0.98 (95% confidence interval (95% CI) 0.96–1.00), *p* = 0.0218). The majority of respondents stated that religious services had become shorter, attended by fewer people and with reduced singing or chanting. Most (869/893, 97.3%) stated their place of worship complied with government guidelines, with 803/887 (90.5%) reported that their place of worship enforced face mask wearing and 793/887 (89.4%) at least moderately happy with precaution measures. Our study demonstrates the significant impact of COVID-19 in places of worship but a high degree of compliance with guidelines. Face masks, despite practical difficulties, appeared to be more acceptable if there was an incentive of being able to sing and chant.

**Supplementary Information:**

The online version contains supplementary material available at 10.1007/s10943-022-01641-2.

## Introduction

The COVID-19 pandemic has led to the widespread adoption of public health measures such as enhanced hand hygiene, universal wearing of face masks and social distancing. (Centres for Disease Control & Prevention, 2021; Public Health England, 2021) In addition, public health authorities have advocated against large gatherings especially in indoor settings, as a means to prevent further outbreaks. This is because large numbers of people meeting together, sometimes for many hours and often in confined spaces, can facilitate transmission of airborne viruses. (Quadri, 2020) Well publicised examples of COVID-19 clusters include the Shincheonji Church of Jesus in South Korea and the Sri Petaling mass gathering in Malaysia, which at one point accounted for more than 60% and 35% of cases in their respective countries, and were the largest clusters of infection within each country. (Kim et al., 2020; Mat et al., 2020) This could partly explain the observation from the UK that religious faith could be associated with increased COVID-19-related mortality, even when adjusted for confounding variables.(Gaughan et al., 2021).

Group singing has been identified as a high-risk activity for transmission of SARS-CoV-2: a choir practice in Washington, USA led to up to 87% of attendees developing COVID-19 from one index case, a so-called super spreading event. (Hamner et al., 2020) Further evidence to support this risk has come from experimental studies: it has been demonstrated that airborne droplets produced during singing do not appear to settle rapidly, and without adequate ventilation, could lead to an outbreak. (Bahl et al., 2020) A second study showed that normal and loud singing produced more aerosol particles, normally defined as 0.5–10 µm, compared to the same volume while speaking, which would increase risk of airborne transmission of viruses. (Alsved et al., 2020) Other aspects of religious practice, such as holy communion and touching of the Torah, may also increase the likelihood of viral transmission. (Anyfantakis, 2020; Hartley et al., 2020).

These risks associated with communal worship and singing led to onsite religious services being halted in England in March 2020 because of the rising incidence of COVID-19, with many services moving online. (Bryson et al., 2020; Modell & Kardia, 2020) Services and gatherings onsite resumed in July 2020 but with a ban on congregational singing and chanting, limits on numbers allowed in congregations, and a requirement for social distancing and the wearing of face masks. (Ministry of Housing, Communities & Local Government, 2021) UK government guidance in April 2021 advocated for singing to be “limited to one person where possible” and stated “communal singing should not take place”, even in the presence of social distancing or usage of face masks. (Ministry of Housing, Communities & Local Government, 2021) However, some relaxation of the rules and singing outdoors did occur in late March 2021. (Burgess, 2021).

It has been noted by Public Health England that “wearing face coverings reduces the mass of aerosol expelled when singing”, although the same report concedes that there is a lack of evidence to suggest the degree to which wearing face masks may reduce the transmission of SARS-CoV-2. (Public Health England, 2020) One study involving 12 singers demonstrated that singing with a surgical face mask reduced the number of generated aerosols to a level similar to normal talking, although this did not reach statistical significance, (Alsved et al., 2020) while wearing face masks could reduce the large variability of droplets being produced when singing (Ho and Davies et al., 2021).

Closure of places of worship during the first lockdown in England and restrictions implemented since then have significantly changed many people’s daily or weekly worshipping routines, affecting their ability to pray, enjoy group discussion or take part in singing or chanting. In particular, permitting singing was seen as important as it may vastly improve congregants’ worshipping experiences, and restore “a sense of celebration.”(Burgess, 2021).

In this study, we recruited practising worshippers of any faith to complete an online questionnaire to improve our understanding of how religious worship has changed during the COVID-19 pandemic in the UK. Our primary aim was to understand how acceptable people would find face mask wearing in places of worship and whether they would be prepared to sing or chant whilst wearing them. Our secondary aims were to understand the changes in worshipping practice due to COVID-19 and how well places of worship have complied with COVID-19-related safety guidelines.

## Methods

### Study Design

CONFESS (COvid aNd FacE maSkS) was a cross-sectional study comprising an online questionnaire to assess the effect of the COVID-19 pandemic on religious practice. Participants were required to be at least 18 years old and be able to understand English. Participation was voluntary and respondents were recruited via a convenience sampling technique using word of mouth, targeted advertising through religious institutions, social media such as Facebook and WhatsApp, and publicity with the British Broadcasting Corporation (BBC), which featured the study in the national news bulletin and a religious affairs programme. (Schraer, 2020) Particular efforts were made to recruit participants from a range of religious backgrounds including Christian, Muslim, Jewish, Hindu and Buddhist by contacting specific groups, such as through Facebook (Supplementary Table 1). In addition, religious leaders were approached directly from churches, mosques, synagogues, and Hindu, Buddhist and Hare Krishna temples. Recruitment and questionnaire completion occurred between August and November 2020, with the last questionnaire completed on 5 November 2020.

### Survey Measures

The CONFESS questionnaire included items concerning demographic characteristics, including age, sex, religion and ethnic background; changes in worshipping practice due to COVID-19; importance of religious life and singing; acceptability and comfort of face mask wearing during communal worship and while singing during worship; awareness and understanding of government guidelines regarding COVID-19 and compliance of participants’ place of worship with these guidelines. The majority of questionnaire items were closed questions. However, open-ended questions regarding face mask acceptability and comfort as well as the impact of COVID-19-related restrictions on communal worship were also included in order to gain deeper insights. It was also possible to skip questions so not all participants answered every question.

### Data Analysis

Quantitative data were analysed using R software version 4.0.4. We performed chi-squared tests for categorical variables, with a *p*-value of ≤ 0.05 taken as statistically significant. We performed univariable and multivariable (adjusted) logistic regression to assess for characteristics which may predict face mask acceptance. We adjusted for sex, age (as a continuous variable), highest level of education, religion, ethnicity, relationship status, place of residence and employment status. As this was a cross-sectional study, we presented both unadjusted and adjusted prevalence odds ratios (OR), with 95% confidence intervals (95% CI), as opposed to prevalence rate ratios, because the variables included in the analysis were long-term characteristics of respondents. In addition, we performed thematic analysis of open-ended questions to complement quantitative analysis. We followed STROBE guidelines in the reporting of this manuscript. (von Elm et al., 2014).

## Results

### Demographics

**Fig. 1:**
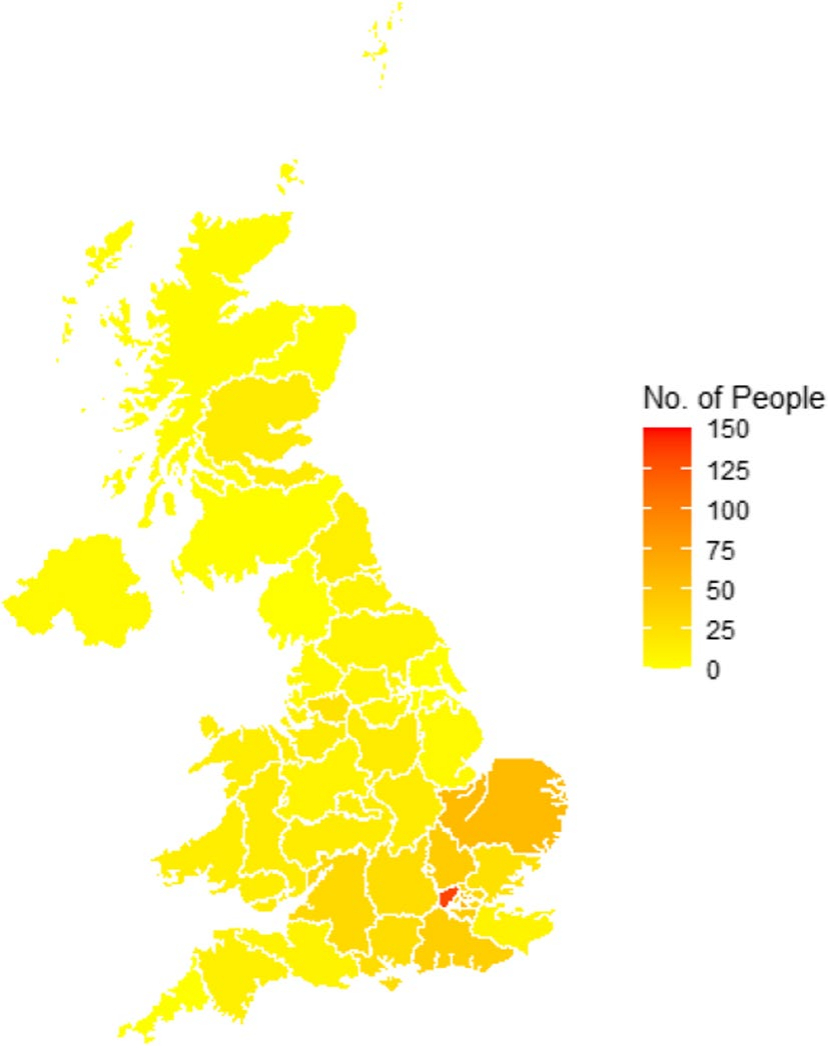
Region of UK residence of CONFESS questionnaire respondents

In total, 1063 people volunteered for the study. A total of 939 (88.3%) participants completed the questionnaire and were included in the analysis. Demographic characteristics of included respondents are shown in Table 1. Median age was 52.7 years and approximately two-thirds were female, while 845 (90.0%) completed at least undergraduate-level education. The majority of participants were Christian (*n* = 758, 80.7%), followed by Jewish (*n* = 145, 15.4%) and of White ethnicity (*n* = 869, 92.5%). Most (789, 84.0%) participants lived in an urban area. Of the 831/939 (88.5%) participants who gave valid postcode data, 305 participants (36.7%) lived in London, while 122 (14.7%) participants lived in East of England and 105 participants (12.6%) lived in South East England (Fig. 1). Most participants (779/939, 83.0%) reported neither had suspected nor confirmed COVID-19 infection previously (Supplementary Table 2).

**Table 1:** Demographic characteristics of survey participants (*n* = 939) and their association with face mask acceptability – if required to wear a face mask when singing or chanting in a place of worship

Characteristic		Participants	Face mask acceptability		Unadjusted odds ratio (95% CI)	Significance (*p*-value)	Adjusted odds ratio (95% CI)	Significance (*p*-value)
			Very/somewhat acceptable	Very/somewhat unacceptable				
Demographic characteristics								
Sex^b^	Female	620 (66.1%)	463/564 (82.1%)	101/564 (17.9%)	–	–	–	–
	Male	318 (33.9%)	209/271 (77.1%)	62/271 (22.9%)	0.74 (0.52–1.05)	0.0905	0.70 (0.47–1.05)	0.0795
Age^c^	Median age (range), years	52.7 (18–85)	–	–	0.98 (0.97–0.99)	0.0050	0.98 (0.96–1.00)	0.0218
	18–34 years	161 (17.7%)	128/140 (91.4%)	12/140 (8.6%)	–	–		
	35–64 years	585 (64.1%)	406/523 (77.6%)	117/523 (22.4%)	0.33 (0.17–0.59)	< 0.001	0.30 (0.13–0.61)	0.0016
	≥ 65 years	166 (18.2%)	119/149 (79.9%)	30/149 (20.1%)	0.37 (0.18–0.74)	0.0070	0.38 (0.14–1.02)	0.0619
Highest educational level	Undergraduate degree or professional qualification	470 (50.1%)	345/422 (81.8%)	77/422 (18.2%)	–	–	–	–
	Postgraduate degree	375 (39.9%)	272/334 (81.4%)	62/334 (18.6%)	0.98 (0.68–1.42)	NS	0.97 (0.65–1.44)	NS
	A-levels or equivalent/post-16 vocational course	72 (7.7%)	42/60 (70.0%)	18/60 (30.0%)	0.52 (0.29–0.97)	0.0345	0.52 (0.28–0.99)	0.0403
	GCSE/CSE/O-levels or equivalent/no qualifications	22 (2.3%)	13/19 (68.4%)	6/19 (31.6%)	0.48 (0.18–1.41)	NS	0.58 (0.21–1.73)	NS
Religion	Christian	758 (80.7%)	559/688 (81.3%)	129/688 (18.8%)	–	–		–
	Jewish	145 (15.4%)	92/119 (77.3%)	27/119 (22.7%)	0.79 (0.50–1.28)	NS	0.88 (0.53–1.49)	NS
	Other^d^	36 (2.8%)	21/28 (75.0%)	7/28 (25.0%)	0.69 (0.30–1.79)	NS	0.61 (0.23–1.78)	NS
Ethnicity	White British	792 (84.4%)	564/707 (79.8%)	143/707 (20.2%)	–	–	–	–
	Other White background	77 (8.2%)	57/69 (82.6%)	12/69 (17.4%)	1.20 (0.65–2.41)	NS	1.27 (0.66–2.60)	NS
	Asian/Asian British	26 (2.8%)	20/23 (87.0%)	3/23 (13.0%)	1.69 (0.57–7.24)	NS	1.53 (0.43–7.54)	NS
	Black/African/Caribbean	12 (1.3%)	9/9 (100%)	0/9 (0.0%)	–	NS	–	NS
	Mixed/Multiple ethnic groups	16 (1.7%)	12/13 (92.3%)	1/13 (7.7%)	3.04 (0.59–55.66)	NS	1.96 (0.35–36.99)	NS
	Other	15 (1.6%)	9/13 (69.2%)	4/13 (30.8%)	0.57 (0.18–2.13)	NS	0.62 (0.19–2.46)	NS
Relationship status	In a relationship/married and cohabiting	686 (73.1%)	495/616 (80.4%)	121/616 (19.6%)	–	–	–	–
	In a relationship/married but living apart	36 (3.8%)	25/30 (83.3%)	5/30 (16.7%)	1.22 (0.50–3.68)	NS	0.89 (0.35–2.78)	NS
	Single, divorced or widowed	60 (6.4%)	38/50 (76.0%)	12/50 (24.0%)	0.77 (0.40–1.59)	NS	0.71 (0.35–1.51)	NS
	Single, never married	157 (16.7%)	114/139 (82.0%)	25/139 (18.0%)	1.11 (0.70–1.83)	NS	0.75 (0.44–1.30)	NS
Place of residence	City/town	789 (84.0%)	563/694 (81.1%)	131/694 (18.9%)	–	–	–	–
	Village/rural dwelling	150 (16.0%)	109/141 (77.3%)	32/141 (22.7%)	0.79 (0.52–1.24)	NS	0.83 (0.52–1.35)	NS
Employment status	Employed (full-time)	374 (39.8%)	273/326 (83.7%)	53/326 (16.3%)	–	–	–	–
	Employed (part-time)	175 (18.6%)	120/158 (75.9%)	38/158 (24.1%)	0.61 (0.38–0.98)	0.0407	0.62 (0.37–1.04)	0.0661
	Self-employed	113 (12.0%)	79/100 (79.0%)	21/100 (21.0%)	0.73 (0.42–1.30)	NS	0.93 (0.51–1.73)	NS
	Retired	193 (20.6%)	139/176 (79.0%)	37/176 (21.0%)	0.73 (0.46–1.17)	NS	0.80 (0.43–1.51)	NS
	Student (university/school)	35 (3.7%)	27/31 (87.1%)	4/31 (12.9%)	1.31 (0.49–4.57)	NS	1.04 (0.33–4.04)	NS
	Other^e^	49 (5.2%)	34/44 (77.3%)	10/44 (22.7%)	0.66 (0.32–1.48)	NS	0.62 (0.28–1.43)	NS

NS, not significant (*p* > 0.10); 95% CI, 95% confidence interval; GCSE, General Certificate of Secondary Education; CSE, Certificate of Secondary Education

^a^Missing data on face mask acceptability: *n* = 78 (8.3%)

^b^The variable sex was defined as sex at birth, which agreed in all cases with current gender identity, where the latter question was completed. One respondent reported that their gender-identity was different from their birth-assigned gender, but sex at birth and current gender identity were the same for this individual. Missing data: n = 1 (0.1%)

^c^Missing data: 27/939 (2.9%)

^d^“Other” category includes Muslim, Buddhist, Hindu, Sikh, any other religion and no religion (numbers of respondents too small to report separately)

^e^“Other” category includes homemaker, full-time parent, carer, job seeker and those unable to work due to disability (numbers of respondents too small to report separately)

### Face Mask Acceptability

Most (872/939, 92.9%) respondents answered questions about the face mask they most commonly wore. Of those who answered, reusable masks (661; 75.8%) and disposable surgical masks (157; 18.0%) were commonest. The majority (861/939, 91.7%) of respondents answered questions on face mask acceptability and comfort; 346 (40.2%) and 326 (37.9%) respondents to the question found it very acceptable and somewhat acceptable, respectively, to be required to wear a face mask and reduce their singing volume to sing or chant safely. While 421 participants (48.9%) reported never having sung in a face mask, most respondents (610, 70.8%) reported having already worn a face mask for at least an hour.

When asked to provide more details regarding their attitudes to face mask wearing for singing during communal worship as an open-ended question, 564 (60.1%) provided details. The overarching response was that face masks were unpleasant, but they were better than not singing at all (quotes 1–3, Table 2). However, there were participants who felt very strongly that they couldn’t sing with a face mask (quotes 4–5). A large number of respondents also expressed concerns about singing volume – whether they could sing more quietly if required (quote 10), and what impact that would have on their enjoyment and spirituality, expressed in terms of restriction and lack of freedom (quotes 14–15).

**Table 2:** Quotations of communal worshippers illustrating the emerging themes

(A) Face mask acceptability during singing	
Acceptable if face mask use allowed communal worshippers to sing during services, despite discomfort	
1	“If the ability to sing is predicated on mask wearing, then what I want is to sing with others, so let’s crack on.”
2	“Any chance to sing would be amazing – even with these limitations!”
3	“I would much rather sing with these restrictions than not sing at all.”
Face masks too great a barrier for singing	
4	“I would not sing while wearing a face mask.”
5	“Singing with a mask on would not work for me.”
Preference for unrestricted singing online rather than masked singing	
6	“Still opting for remote singing over masked.”
7	“We prefer not to attend as the mask wearing and inability to sing together is off putting. We prefer to stay at home and sing as loud as we like with no masks!”
Face masks distracting / decreasing expression	
8	“The mask would be distracting and my focus would be changed from worshipping God to feeling distracted by a face covering.”
9	“Singing and praying together as a community is an integral part of why we worship together. As well as being physically uncomfortable and difficult to focus it creates barriers… we become individuals with no personality, not a community.”
(B) Acceptability of reducing singing volume	
Acceptable	
10	“I don’t know if I *can* sing quietly but will have to try!”
11	“Quiet singing is far better than no singing, the spiritual words are the most important part of our worship.”
Somewhat unacceptable	
12	“It was lovely to be part of a socially distanced service but the need to minimise volume of prayer and singing restricted the joy and sense of community.”
13	“It's better than not singing at all, but I would prefer to be able to sing freely.”
Not acceptable	
14	“There's no point in singing if you can't do it wholeheartedly.”
15	“Singing LOUDLY is the best part about singing at church.”
(C) Singing as an expression of freedom	
16	“I hate the idea of anyone singing into a mask. Singing is about freedom.”
17	“There is usually a freedom in singing which would be affected by these restrictions.”
(D) Face mask comfort	
Steaming up glasses	
18	“Main issue is not being able to wear my glasses as they mist up and I can't see the words without them”
19	“Despite following the recommendations for glasses users, my glasses still steam up, if I speak or sing, they steam up even more.”
Breathing difficulties in general	
20	“It restricts my ability to breathe and be heard slightly”
21	“I get too hot and sometimes feel I am not getting enough oxygen.”
Asthma	
22	“I have asthma, so wearing a face mask is very difficult, and speaking with it even more”
23	“I find that occasionally it becomes more of an effort to breathe, and if this happens I need to remove the mask for a couple of minutes. I am asthmatic.”
Breath intake while singing	
24	“When singing the mask sucks in against my mouth”
Hot/sweaty	
25	“Don't like the confinement, stuffiness, impaired contact / expression.”
26	“I get sweaty and hot if I talk, singing is worse!”
Sore ears	
27	“The face masks are relatively comfortable but I get quite hot and they hurt my ears (I wear glasses) if wearing for a long time.”
28	“I feel smothered, my ears are sore and all this distracts me during worship.”
Face mask movement	
29	“A face mask… feels like a barrier in my worship to God. I need to continually think about it moving.”
30	“Masks can tend to slip when mouth movements are made.”
Face masks distracting from worship	
31	“Church services have been reduced in content, length and numbers, and I have been attending less because of the distress caused by being forced to wear a face covering.”
Communication impairment	
32	“Mask makes communication more difficult.”
33	“I can't hear other people when they speak wearing one (as I obviously use mouth signals as well as auditory signals in order to hear).”
34	“I find wearing a face mask frustrating as I miss face expressions and they ride up when you speak/sing.”
Impairment of religious expression	
35	“From the point of view of worship—there is a verse in 2 Corinthians (2:18) that speaks of our 'unveiled faces' reflecting the Lord's glory. While I FULLY accept (and comply with) the need to wear face masks to protect against Covid-19 transmission, at a deep level I feel there is something about wearing a mask that makes my relationship with God in worship less open. I also think in human interaction it somehow de-personalises us and makes communication with one another less 'open'.”
36	“A mask is restrictive for worship and communication.”
Discomfort increases with duration of use	
37	“Discomfort increases with time, feeling hot and 'steamed-up'”
38	“The length of time I need to wear a mask for affects how comfortable I feel. It becomes more uncomfortable the longer I wear it.”
Discomfort decreases with breaks in use	
39	“The mask is less than ideal but I get used to it pretty quickly, as long as I can take it off from time to time.”
Discomfort decreases with frequency of use	
40	“I find the more I wear the mask in different situations, the more comfortable I get with it.”
41	“Wearing a mask is not particularly comfortable but I have got used to it so it no longer bothers me. I was surprised that it felt OK to sing in a mask but found that it tends to move around and you suck the material in when you breath. I have recently purchased a singer’s mask which has more space and fits really securely and is much more comfortable for singing.”
Fewer issues for respondents reporting occupational use of face masks	
42	“I wear a surgical face mask for healthcare work purposes every weekday—very familiar and comfortable with it
43	“I work in the NHS therefore am used to wearing a mask on a regular basis.”

We also assessed participant characteristics which may affect acceptability of face masks (Table 1). Univariable and multivariable regression indicated very few predictors of congregants finding face mask use acceptable. We found that increasing age was associated with a lower likelihood of face mask acceptability, both when evaluating age as a continuous variable (unadjusted OR (uOR) for each additional year of age: 0.98 (95% CI 0.97–0.99) *p* = 0.0050, adjusted OR (aOR): 0.98 (95% CI 0.96–1.00), *p* = 0.0218) and as categories (aOR for 35–64 years compared to 18–34 years: 0.30 (0.13–0.61) *p* = 0.0016; aOR for ≥ 65 years compared to 18–34 years: 0.38 (0.14–1.02) *p* = 0.0619 (borderline significant)). Age categories used in Table 1 were defined a priori, but stratifying by narrower, 5-year categories suggested that participants aged less than 40 were more likely to be accepting of face masks compared to participants aged 40 and over (*Χ*^2^ = 12.47, *p* = 0.0004).

In addition to younger age, we found that people educated to A-level and vocational standard were more accepting of face masks compared to those who had received undergraduate education or higher (uOR: 0.52 (95% CI 0.29–0.97) *p* = 0.0345, aOR: 0.52 (95% CI 0.28–0.99), *p* = 0.0403). Furthermore, there was a trend that men found face mask wearing with quieter singing less acceptable than women (uOR: 0.74, 95% CI 0.52–1.05, *p* = 0.0905, aOR: 0.70, 95% CI 0.47–1.05, *p* = 0.0795), although this did not reach statistical significance. We also assessed if having had suspected or confirmed COVID-19 would predict face mask acceptability but this was not significant (uOR: 0.76, 95% CI 0.50–1.19, *p* = 0.2201).

### Face Mask Comfort

**Fig. 2:**
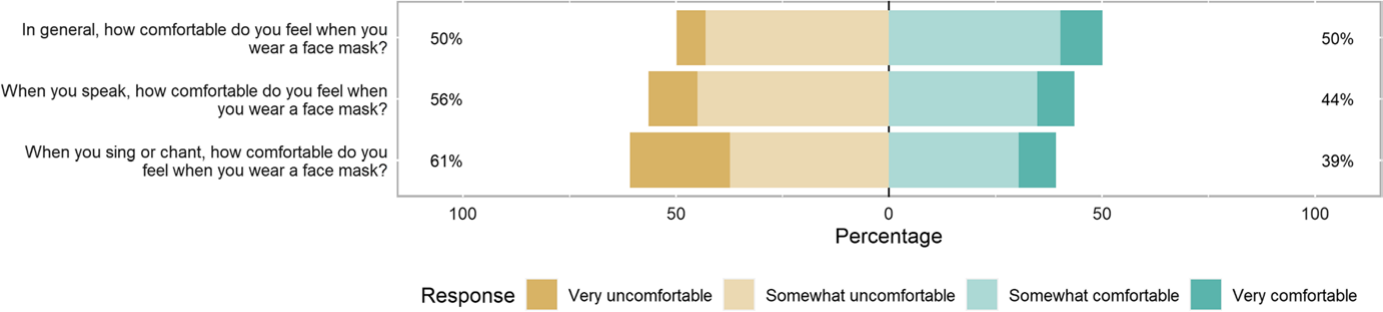
Face mask comfort in general, during speaking and singing/chanting

Just under half of respondents (428/858, 49.9%) found wearing face masks in general somewhat or very uncomfortable, but increasing numbers found it uncomfortable wearing face masks for speaking and for singing (Fig. 2). When asked to provide more details on how comfortable the respondent feels when wearing a face mask while singing/chanting, 564/861 (65.5%) provided a response. Respondents frequently raised similar issues with face mask-wearing (Table 2), especially practical problems such as glasses steaming up (quotes 18–19) and breathing being less comfortable (quotes 20–21). Face masks were particularly difficult for asthma sufferers (quotes 22–23). In addition, many reported that face masks inhibit their ability to communicate properly, particularly for those reliant or partly reliant on lip reading (quotes 32–34). However, it appeared that face mask comfort improved with increasing use (quotes 40–41) and was better tolerated in people who worked in occupations which required their use (quotes 42–43).

### Changes in Places of Worship During the Pandemic

**Fig. 3:**
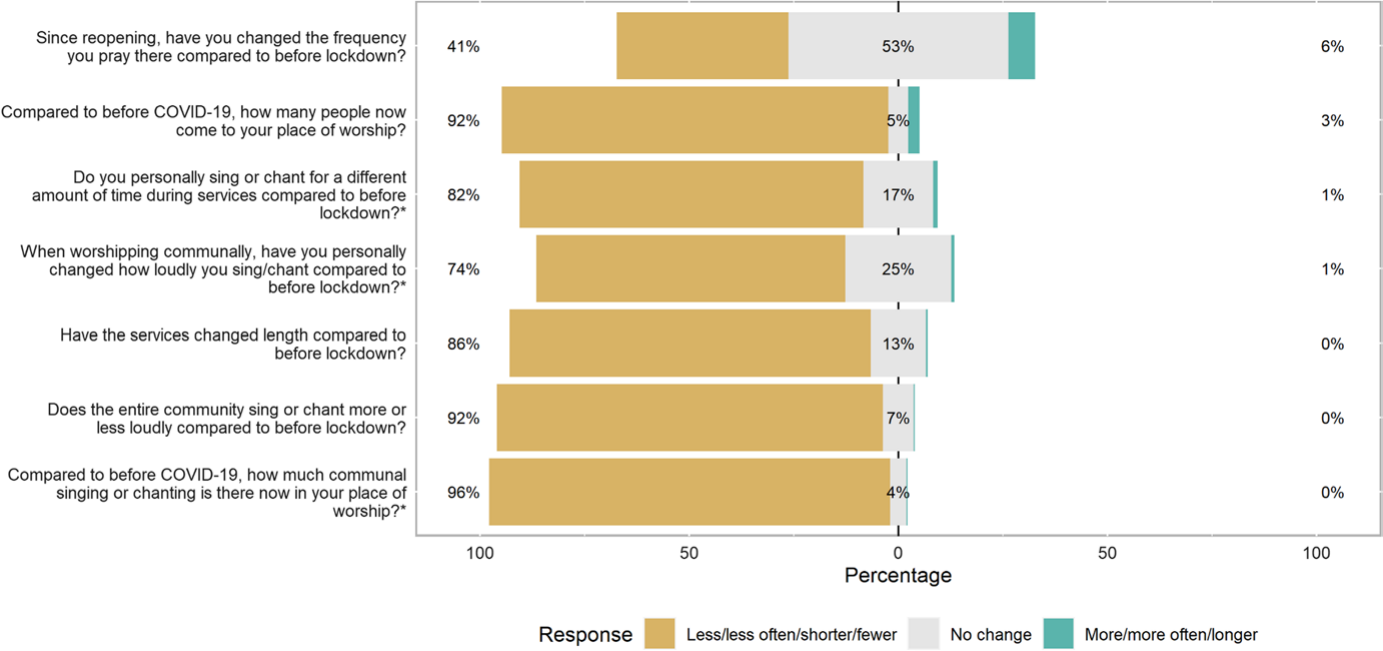
COVID-19-related changes to place of worship, comparing post-first UK lockdown with pre-first UK lockdown. *Respondents reporting no singing, personally or communally, are not shown

Our respondents reported substantial changes in communal worship post-lockdown. Fewer numbers of people were in attendance, services were shorter, with reduced frequency or no singing/chanting, both on a communal and personal level (Fig. 3). The notable exception to this was change in frequency of attendance, with over half of respondents reporting no change in their frequency of prayer at their place of worship, although it was unclear whether this represented private prayer or communal worship. The vast majority of participants reported that religious faith was important to them (858/907; 94.6%), and so while fewer congregants were attending services post-lockdown, study respondents may be more willing to attend worship in person than congregants in general.

Most places of worship were reported to be complying with COVID-19-related restrictions: 869/893 (97.3%) reported being aware of COVID-19-related rules in place at their place of worship, with 624/659 (94.7%) and 641/659 (97.3%) reporting that congregants are moderately or very careful to adhere to social distancing and continuously wearing face masks. A large majority (803/887; 90.5%) reported that their place of worship enforces face mask-wearing rules and that they were either very or moderately happy with the precautions currently in place (793/887; 89.4%) (further details in Supplementary Table 3).

### Role of Religion and Music

**Fig. 4:**
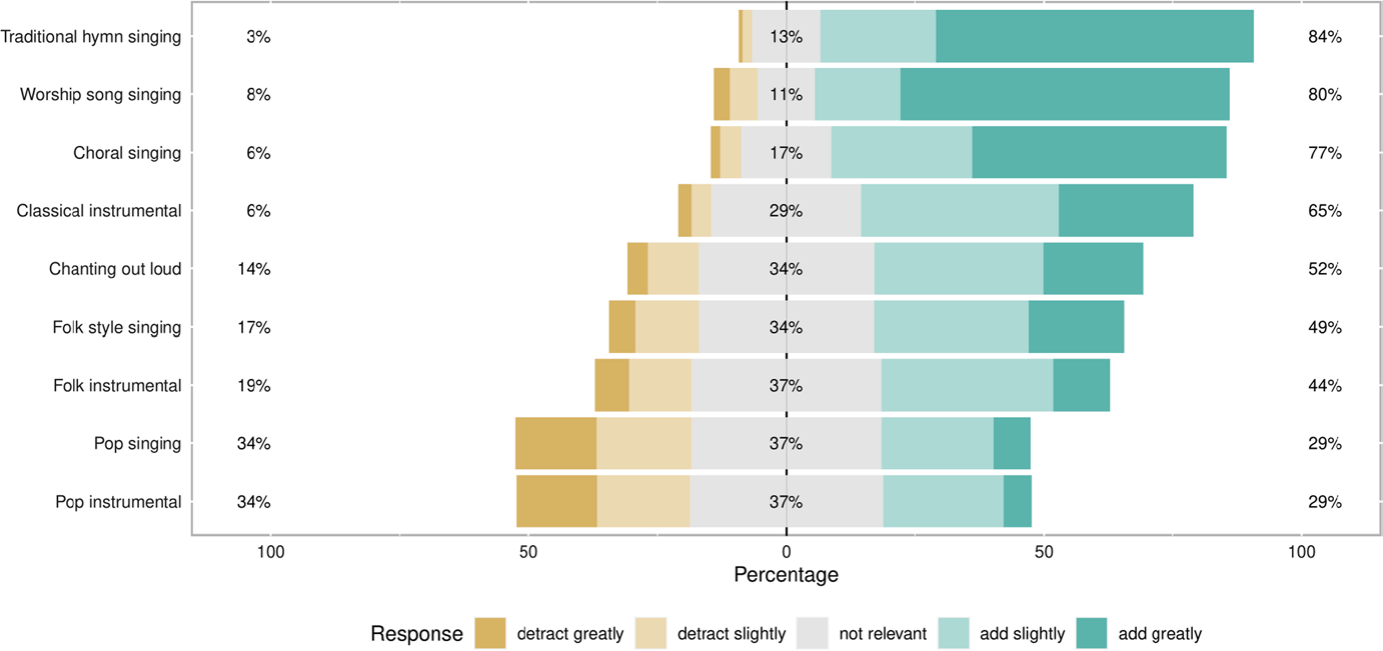
Study respondents’ views on how different musical styles affect their religious experience

We were also interested in the role of music and how it may affect an individual’s religious experience (Fig. 4). The vast majority of respondents found traditional hymn singing, worship song singing and choral singing most enhanced their religious experience. This contrasted to pop singing and pop instrumental, which were generally felt to be more distracting.

## Discussion

Our study is one of the largest to look at the issue of face masks in the context of religion and worship and the first in the era of COVID-19. We have demonstrated that more than half of our respondents found wearing face masks uncomfortable when speaking or singing. They often had practical difficulties such as having sore ears, face mask slippage or steamed-up glasses and these issues became more troublesome with age. We also found that younger age and A-level and vocational level education compared to undergraduate education were predictive of better acceptability of face masks. The latter result is perhaps surprising and could be purely due to chance, but certain vocational occupations such as construction may also require routine wearing of face masks and may explain this observation. We noted a large majority of our population were willing to trade comfort for the ability to sing; 78.1% were prepared to wear face masks and reduce the volume of their singing in order to resume singing or chanting during communal worship. Most of the communal religious worshippers responding to our questionnaire were already used to wearing a face mask for at least an hour. It is likely that since our study started 6 months after the first national lockdown in the UK, attitudes to face masks had started to shift and become part of routine life in the COVID-19 era. In addition, participants may have had fatigue from restrictions on daily life and may be more likely to compromise to return to some semblance of normality. This may be especially true for singing where there is overwhelming evidence to suggest that it enhances worship. Interestingly, we also noted that respondents already accustomed to wearing face masks such as healthcare workers reported fewer issues with wearing face masks in places of worship.

We also noted a polarisation of opinion regarding face masks: some believed wearing a face mask was too great a barrier and would not be prepared to sing using one, feeling that it interfered with freedom of religious expression. In contrast, the majority felt that despite the discomfort, it was worth using them to enable worship and singing to continue, although some respondents found them distracting and could be sucked into the mouth.

Previous research in this area is limited, although a cluster-randomised trial did not demonstrate a difference in face mask wearing in the transmission of respiratory viruses during Hajj.(Alfelali et al., 2020) However, it should be noted that daily face mask use was low (25% in the intervention group). (Alfelali et al., 2020) Reasons given for non-usage of face masks included difficulty in breathing (26%) and discomfort (22%). (Alfelali et al., 2020) Several other studies on Hajj pilgrims have cited similar concerns over the non-usage of face masks, although compliance with face masks increased with increasing perception of effectiveness. (Alqahtani et al., 2015, 2016) However, since the start of the COVID-19 pandemic, use of face masks has become widespread and in certain scenarios mandatory, hence these previous findings may be less comparable to our results where overall there was good compliance.

It is worth mentioning that face mask wearing has sometimes been a contentious issue. While there has been generally good population-level compliance, there have been anti-mask rallies around the world, sometimes associated with violence. (Taylor & Asmundson, 2021) The reasoning has often been multifactorial but includes discomfort, belief that they are ineffective and violation of civil liberties. (Taylor & Asmundson, 2021) Negative attitudes to face mask use have also been associated with conservative political views. (Taylor & Asmundson, 2021) A study from the US suggested that greater religiosity led to reduced adherence to policies to stay at home and reduce social contacts, as it was felt to impinge on personal and religious freedom. (DeFranza et al., 2020) However, although several participants mentioned face mask use led to reduced freedom of worship and religious expression, negative attitudes to face mask use and compliance did not appear to be widely held in our cohort.

Finally, our study found that there was good compliance with government guidelines by places of worship in the UK, as well as good overall compliance by congregants. One participant remarked: “I would like to add that of all the places I have been to since the start of COVID-19 my church has by far treated social distancing and disinfecting most seriously. In fact, I feel that going to church has made me more careful because our priest encourages us to abide by the government's recommendations.” There was a high degree of satisfaction with prevention measures, with 89.4% very happy or moderately happy with precautions. This would suggest that any relaxation of COVID-19 guidelines to allow some level of singing is likely to be enforced well. It would be worthwhile in future studies to assess how much influence both religious and public health leadership had in encouraging compliance with government guidelines within this cohort.

There are potentially additional advantages to relaxing restrictions on singing. For example, collective singing has been used to boost morale in Italian cities during lockdown. (Corvo & De Caro, 2020) A step towards normality may also bring about wider benefits. Religion helps to create a sense of belonging and can help foster a sense of connection and attachment, reduce feelings of social isolation and improve mental health. (Dutra & Rocha, 2021; Hathaway, 2020) Religion has been used as a coping mechanism for survival, allowing for a sense of security and hope. (Kowalczyk et al., 2020) Furthermore, religious organisations can play a role in health promotion or provision of welfare, and religious leaders are often seen as pillars of a community, acting as gatekeepers to marginalised or difficult-to-reach communities. (Barmania & Reiss, 2020; Modell & Kardia, 2020) Improving the experience of congregants and allowing for improved engagement with worship may allow religious organisations to have a greater impact on the communities they serve.

### Limitations

The largest limitation to our study is the representativeness of our sample. Despite attempts to maximise inclusivity, our sample was biased towards worshippers from London and the South East, of White ethnicity, with university-level education and predominantly of Christian, and to a lesser extent Jewish, faith. For speed and ease, as recruitment spanned just over 3 months, we used a convenience sampling technique, relying on word of mouth and the researchers’ local networks to recruit as many participants as possible in the short time. Certain religious groups were underrepresented in our study. For example, while 4.8% of the population in England and Wales identify as Muslim, they only accounted for 0.6% of our study. (Office for National Statistics, 2020) In contrast, 0.5% of the England and Wales population identify as Jewish, although they accounted for 15.4% of our respondents. (Office for National Statistics, 2020). These caveats mean that our results are less generalisable to the UK population. Our study was retrospective in design; some questions may be subject to recall bias. The survey was also only available online and shared via digital means, with only English language offered, so participants who do not have access to technology or who are not proficient in English would have been excluded.

## Conclusions

Our study, one of the largest to date, demonstrates the profound impact the COVID-19 pandemic has had on religious worship. Our results suggest that there is good adherence to COVID-19 guidelines in places of worship and there is a real hunger for a return to normal worship with singing and chanting, even if it means additional mitigation measures such as wearing a face mask. In addition, we have identified that face mask discomfort was associated with certain health conditions which increase with age, such as wearing glasses. Improvements in face mask design may help to circumvent these issues.

We believe our work can help to inform the debate in achieving the right balance between ensuring a good worship experience for congregants and reducing the risk of transmission of COVID-19. There could be implications in helping to shape any future update to the government’s COVID-19 policy around communal worship and singing.

## Supplementary Information

Below is the link to the electronic supplementary material.

Supplementary file1 (DOCX 17 KB)

## Data Availability

The full dataset will not be shared but key variables, including the full set of open-ended responses, and not restricted to those used in the preparation of this manuscript, will be made available on reasonable request. Researchers who provide a methodologically sound proposal may access the full dataset after de-identification, directed to Professor Laurence Lovat: l.lovat@ucl.ac.uk. Data requestors will need to sign a data access agreement.

## Code Availability

Available on request to corresponding author.
