# Supplementary material for: Face Mask Acceptability for Communal Religious Worship During the COVID-19 Pandemic in the United Kingdom: Results from the CONFESS Study
Source: J Relig Health. 2022 Aug 24;62(1):608–26. doi: 10.1007/s10943-022-01641-2 (PMC9401196; doi:10.1007/s10943-022-01641-2)
Supplement: Supplementary file 1 — Supplementary file1 (DOCX 17 KB) [file 10943_2022_1641_MOESM1_ESM.docx]

**Supplementary Table 1**: Facebook groups on which the CONFESS Study was advertised.

| Acton Masjid | Babul Murad Centre | Bristol Hindu Temple | Buddhapadipa Temple |
| --- | --- | --- | --- |
| East London Mosque | Friendly Frummers | Hindu Temple Newcastle | Ilford Hindu Temple |
| Iskon Temple Watford | Islamic Integration Community | Jalaram Mandir Temple | Jamia Masjid |
| Jamyang Buddhist Centre | Jewish Connections | Junior Anglican Evangelical Conference | Kingsbury Buddhist Temple |
| London Asian Seventh-Day Adventist Church | London International Christian Church | London International Church @RhemaFaithMinistries | Mayfair Islamic Centre |
| Methodist Central Hall Westminster | MOO Modern Open Orthodox | Moslem Ali Khan Welfare Foundation | Slough Hindu Temple |
| Southampton Hindu Temple | Swindon Hindu Temple | Synagogues of London and the UK | UK Christian Events |
| UK Methodists | UKEvents.net | Vedic Society Hindu Temple |  |
| In addition, we advertised to various large mosques and synagogues around London and the UK | | | |

**Supplementary Table 2:** COVID-19 related characteristics of study participants.

|  |  | **Participants (%)** |
| --- | --- | --- |
| **COVID-19 related characteristics of respondents** | | |
| **COVID-19 infection status** | Yes diagnosed and recovered | 27 (2.9%) |
|  | Yes diagnosed and still ill | 3 (0.3%) |
|  | Not formally diagnosed but suspected | 130 (13.8%) |
|  | Not that I know of / No | 779 (83.0%) |
| **Ever had a SARS-CoV-2 virus swab test** | Yes – positive result | 9 (1.0%) |
|  | Yes – negative result | 274 (29.3%) |
|  | No swab test | 653 (69.8%) |
| **Ever had a SARS-CoV-2 antibody test** | Yes – positive result | 33 (3.5%) |
|  | Yes – negative result | 72 (7.7%) |
|  | No swab test | 834 (88.8%) |
| **Current isolation status** | 1. Living life as normal | 128 (13.6%) |
|  | 2. Not "staying at home" but cut down on usual activities as a precaution | 471 (50.2%) |
|  | 3. Not "staying at home" specifically, but working from home | 132 (14.1%) |
|  | 4. "Staying at home" but not high risk - worried about spreading to others or getting ill | 31 (3.3%) |
|  | 5. "Staying at home" to protect a family member/friend/housemate with an existing medical condition/ high risk | 21 (2.2%) |
|  | 6. "Staying at home" – existing medical condition or categorised as high risk | 23 (2.4%) |
|  | 7. "Self-isolating" – avoiding contact with all people as much as possible due to a COVID-19 diagnosis or possible COVID-19 infection | 0 (0.0%) |
|  | 8. "Staying at home" – ordered government/local authority as part of a lockdown | 28 (3.0%) |
|  | 9. “Staying at home" for a non-COVID-19-related reason e.g., a pre-existing health condition or disability | 4 (0.4%) |
|  | *Missing* | *101 (10*.8*%)* |

**Supplementary Table 3:** Compliance of places of worship and congregants with COVID-19-related restrictions. (Many respondents did not answer these questions if they had not visited their place of worship since the start of the COVID-19 pandemic).

|  |  | **Participants (%)** |  |  | | **Participants (%)** | |
| --- | --- | --- | --- | --- | --- | --- | --- |
| **Compliance of place of worship with government guidelines** | | | **Compliance/satisfaction of congregants with government guidelines** | | | |  |
|  |  |  | **Aware of COVID-19-related** | Yes | 869 (97.3%) | |  |
|  |  |  | **rules for places of worship** | No | 24 (2.7%) | |  |
| **Hand sanitiser** | Available | 825 (87.9%) | **Use of hand sanitiser by** | All | 402 (61.0%) | |  |
|  | Not available | 24 (6.1%) | **congregants upon arrival** | Most | 228 (34.6%) | |  |
|  | Don’t know | 57 (6.1%) |  | About half | 17 (2.6%) | |  |
|  |  |  |  | A few | 11 (1.7%) | |  |
|  |  |  |  | None | 1 (0.2%) | |  |
| **Social distancing** | Required | 850 (95.8%) | **Congregants’ care to social** | Very careful | 400 (60.7%) | |  |
|  | Not required | 2 (0.2%) | **distance** | Moderately careful | 224 (34.0%) | |  |
|  | Don’t know | 35 (3.9%) |  | Slightly careful | 32 (4.9%) | |  |
|  |  |  |  | Not at all careful | 3 (0.5%) | |  |
| **Face mask wearing** | Enforced | 803 (90.5%) | **Congregants’ care** | Very careful | 546 (82.9%) | |  |
|  | Not enforced | 30 (3.4%) | **continuously to wear** | Moderately careful | 95 (14.4%) | |  |
|  |  |  | **face masks** | Slightly careful | 9 (1.4%) | |  |
|  | Don’t know | 54 (6.1%) |  | Not at all careful | 9 (1.4%) | |  |
| **Air flow from outside (e.g.,** | Yes | 630 (71.0%) | **Happy with COVID-19** | Very happy | 559 (63.0%) | |  |
| **open doors, windows)** | No | 87 (9.8%) | **prevention precautions** | Moderately happy | 234 (26.4%) | |  |
|  | Don’t know | 170 (19.2%) | **at place of worship** | Slightly happy | 51 (5.7%) | |  |
|  |  |  |  | Not at all happy | 43 (4.8%) | |  |
